# Does women’s mobile phone ownership matter for health? Evidence from 15 countries

**DOI:** 10.1136/bmjgh-2020-002524

**Published:** 2020-05-17

**Authors:** Amnesty E LeFevre, Neha Shah, Jean Juste Harrisson Bashingwa, Asha S George, Diwakar Mohan

**Affiliations:** 1 Faculty of Health Sciences, University of Cape Town, Observatory, Western Cape, South Africa; 2 International Health, Johns Hopkins University Bloomberg School of Public Health, Baltimore, Maryland, USA; 3 Computational Biology Division, Faculty of Health Sciences, University of Cape Town, Cape Town, South Africa; 4 School of Public Health, University of the Western Cape, Bellville, South Africa

**Keywords:** public health

## Abstract

Mobile phones have the potential to increase access to health information, improve patient–provider communication, and influence the content and quality of health services received. Evidence on the gender gap in ownership of mobile phones is limited, and efforts to link phone ownership among women to care-seeking and practices for reproductive maternal newborn and child health (RMNCH) have yet to be made. This analysis aims to assess household and women’s access to phones and its effects on RMNCH health outcomes in 15 countries for which Demographic and Health Surveys data on phone ownership are available. Multilevel logistic regression models were used to explore factors associated with women’s phone ownership, along with the association of phone ownership to a wide range of RMNCH indicators. Study findings suggest that (1) gender gaps in mobile phone ownership vary, but they can be substantial, with less than half of women owning mobile phones in several countries; (2) the gender gap in phone ownership is larger for rural and poorer women; (3) women’s phone ownership is generally associated with better RMNCH indicators; (4) among women phone owners, utilisation of RMNCH care-seeking and practices differs based on their income status; and (5) more could be done to unleash the potential of mobile phones on women’s health if data gaps and varied metrics are addressed. Findings reinforce the notion that without addressing the gender gap in phone ownership, digital health programmes may be at risk of worsening existing health inequities.

**Summary box**

- Gender inequality exists in mobile phone ownership, and particularly affects poor and rural women.
- Ownership of mobile phones by women is more likely to impact reproductive maternal newborn and child health interventions that are provided by outreach efforts (family planning and child immunisations) than facility-based services (skilled birth attendance, antenatal care 4+).
- Unless gaps in women’s ownership and use of mobile phones is better understood and addressed, digital health programmes may be at risk of worsening existing health inequities.

## Introduction

By 2020, there will be an estimated 7.26 billion mobile phone users worldwide—a figure just short of the global population of 7.58 billion people.^1^ This has transformed phone connectivity and also access to the internet. In low-income and middle-income countries, mobile phones are the primary means of internet access and nearly half of women access the internet using mobile phones.^2^ Increasing access to mobile phones therefore makes digital interventions for health particularly enticing and strategic; to increase access to health information and also to support communication between patients and healthcare providers, and improve the uptake, content and quality of health services. Yet significant gaps persist between men and women’s ownership, access to, and usage of phones which may exacerbate existing inequalities and limit the potential reach and effectiveness of technology use for health improvement.^3^

Women’s phone access, in particular, is highlighted for its transformative and emancipatory potential to accelerate social and economic development.^4–6^ Given existing gender inequalities in many low-income and middle-income countries, mobile phones can be an equaliser—allowing men and women to access the same information.^7^ Mobile phones can help to empower women; connecting them to family, friends, information and services.^2 8^ In doing so, women’s access to mobile phones can challenge power relations and social norms, increasing women’s awareness and autonomy.^9^ In some settings, this may inadvertently increase risk to women; making them targets for backlash in contexts where conservative gender ideologies dominate.^9^

In the health domain, mobile phones are also increasingly becoming a modality for facilitating women’s access to health information and engagement with the health system. While evidence directly linking women’s phone ownership to changes in changes in reproductive maternal newborn and child health (RMNCH) outcomes is limited, secondary analyses of data from Burkina Faso suggest that women’s phone ownership may be associated with improvements in reported modern contraceptive use.^10^ Similar analyses carried out in India suggest women’s phone ownership in urban areas was positively associated with skilled birth attendance, postnatal care and use of modern contraceptives.

Indirectly, women’s phone ownership and access may influence participation in digital health programmes, including mobile health (mHealth) programmes which have been shown to increase women’s decision-making, social status and access to health resources, as well as influence gender relations in positive ways; providing new modalities for health communication and cooperation, and enabling greater male participation in health areas typically targeting women.^6 11^ Mobile phones are also increasingly being used in other ways that give women a voice in the health system, whether through feedback on the content and quality of health services,^12–15^ or through participation in phone surveys used to generate evidence on disease burden^16 17^ or on women’s experiences with the health system.^18^

**Figure 1:**
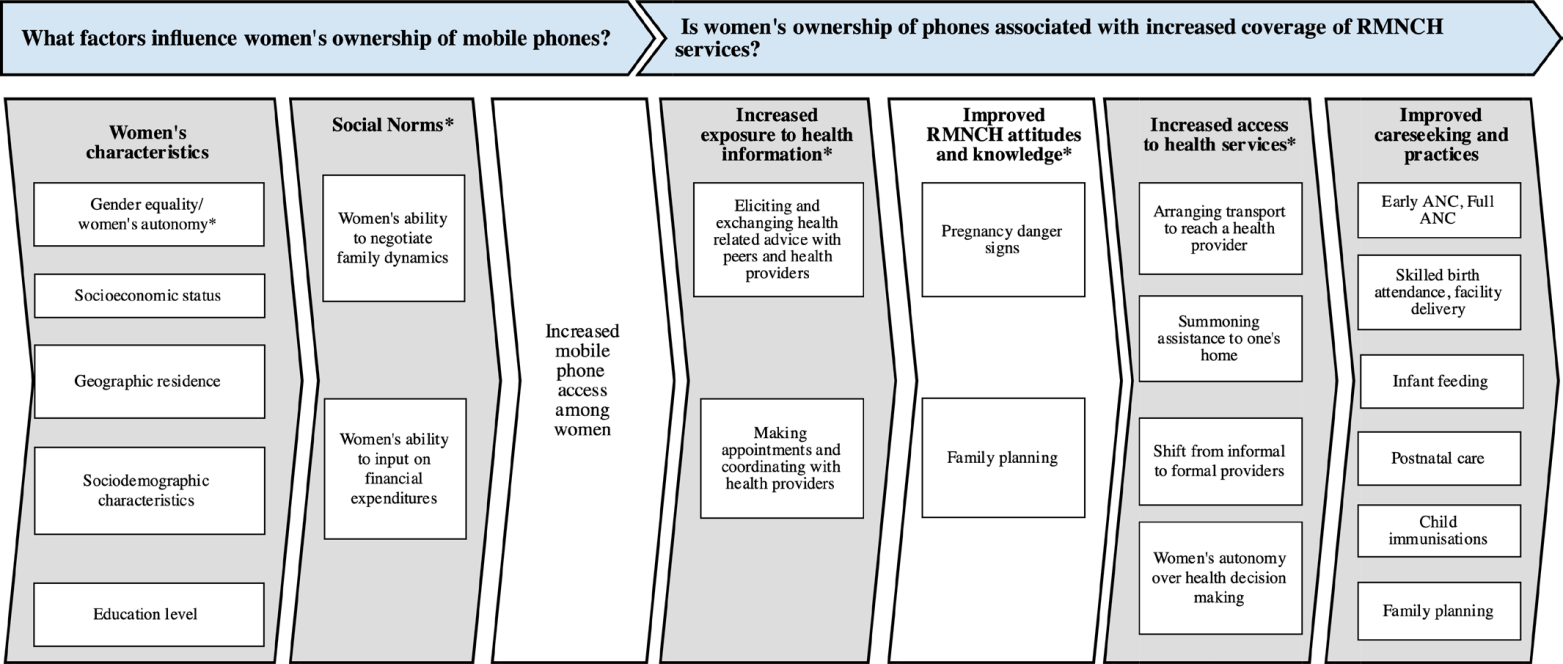
Conceptual framework.

Despite their immense potential, underlying inequities in women’s phone ownership, access and use may inadvertently drive inequities in women’s access to health information and services and in the adoption of practices linked to improved health outcomes, particularly among mothers and children.^9 19 20^ In this analysis, we argue that addressing the gap between men and women’s phone ownership (hereafter referred to as the gender gap) and increasing women’s access to and use of mobile phones has great potential for improving RMNCH health, particularly for populations most left behind tracked by Countdown 2030. In our conceptual framework, we map the relationship between women’s characteristics and social norms on access to phones. With access to a phone, women may have increased exposure to health information including health-related advice, appointment reminders, and/or provider interaction and engagement (figure 1). This, in turn, may yield changes in attitudes and knowledge which drive demand for health services and leads to changes in care-seeking and practices for RMNCH.

Drawing on data available on phone ownership in Demographic and Health Surveys (DHS), analyses will demonstrate that (1) gender gaps in mobile phone ownership vary, but they can be substantial, with less than half of women owning mobile phones in several countries; (2) the gender gap in phone ownership is larger for rural and poorer women; (3) women’s phone ownership is generally associated with better RMNCH indicators; (4) among women phone owners, utilisation of RMNCH care-seeking and practices differs based on their income status; and (5) more could be done to unleash the potential of mobile phones on women’s health if data gaps and varied metrics are addressed. Methods underpinning analyses are described in online supplementary file section 1.

10.1136/bmjgh-2020-002524.supp1 Supplementary data

## Gender gaps in mobile phone ownership vary, but they can be substantial, with less than half of women owning mobile phones in several countries

**Figure 2:**
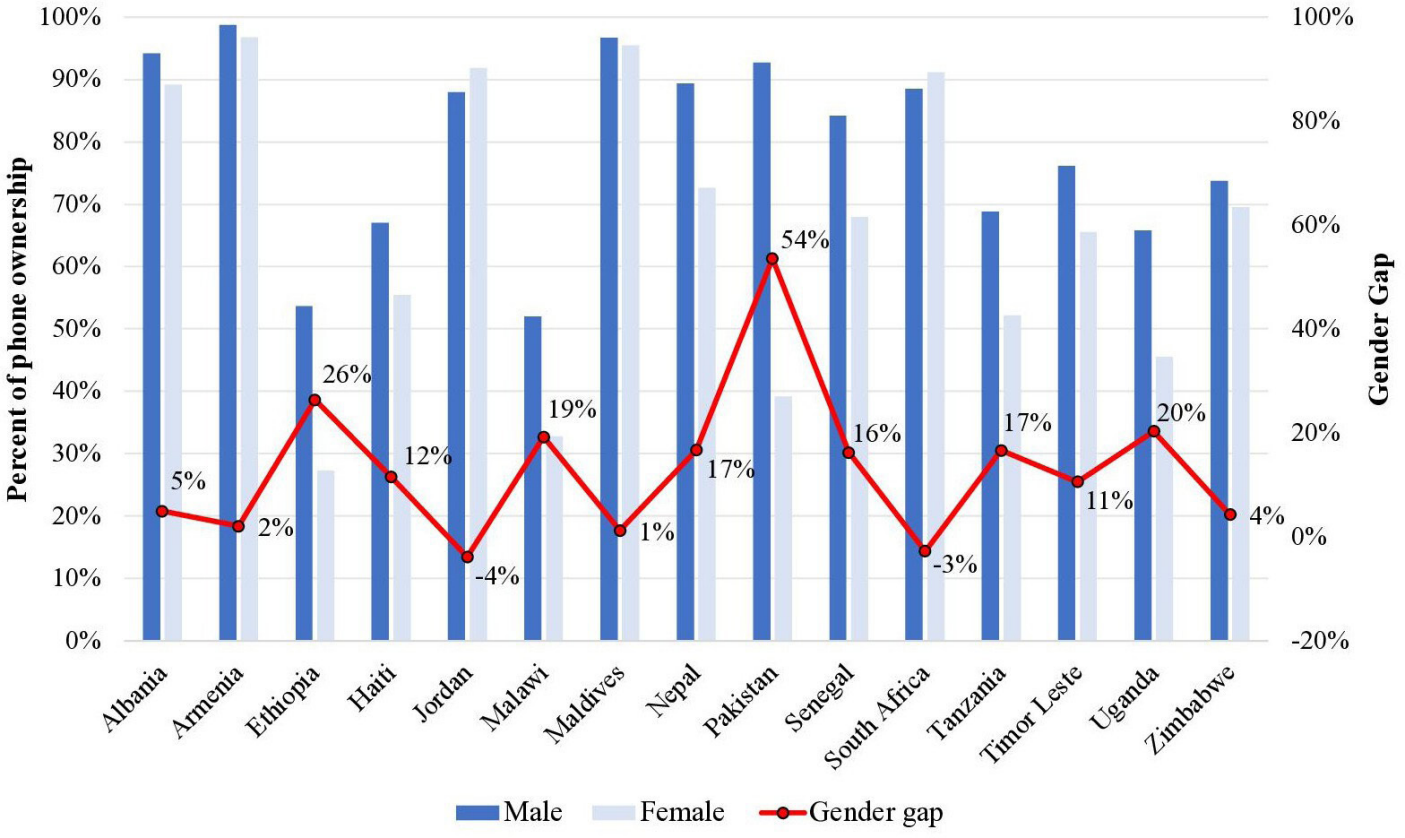
Gender gap in phone ownership across 15 countries.

Data on phone ownership are presented for the 15 countries that have captured DHS data on men’s ownership of mobile phones and the 17 countries that have captured data on women’s ownership of mobile phones since 2015. While the extent of the gender gap in mobile phone ownership varies across countries, it is substantial in several instances (figure 2). In particular, the gender gap in mobile phone ownership is greatest in Pakistan (56%), Ethiopia (25%) and Nepal (24%). In several countries, women’s mobile phone ownership is less than 50% (Ethiopia, Malawi, Pakistan and Uganda).

## The gender gap in phone ownership is worse for rural and poorer women

**Figure 3:**
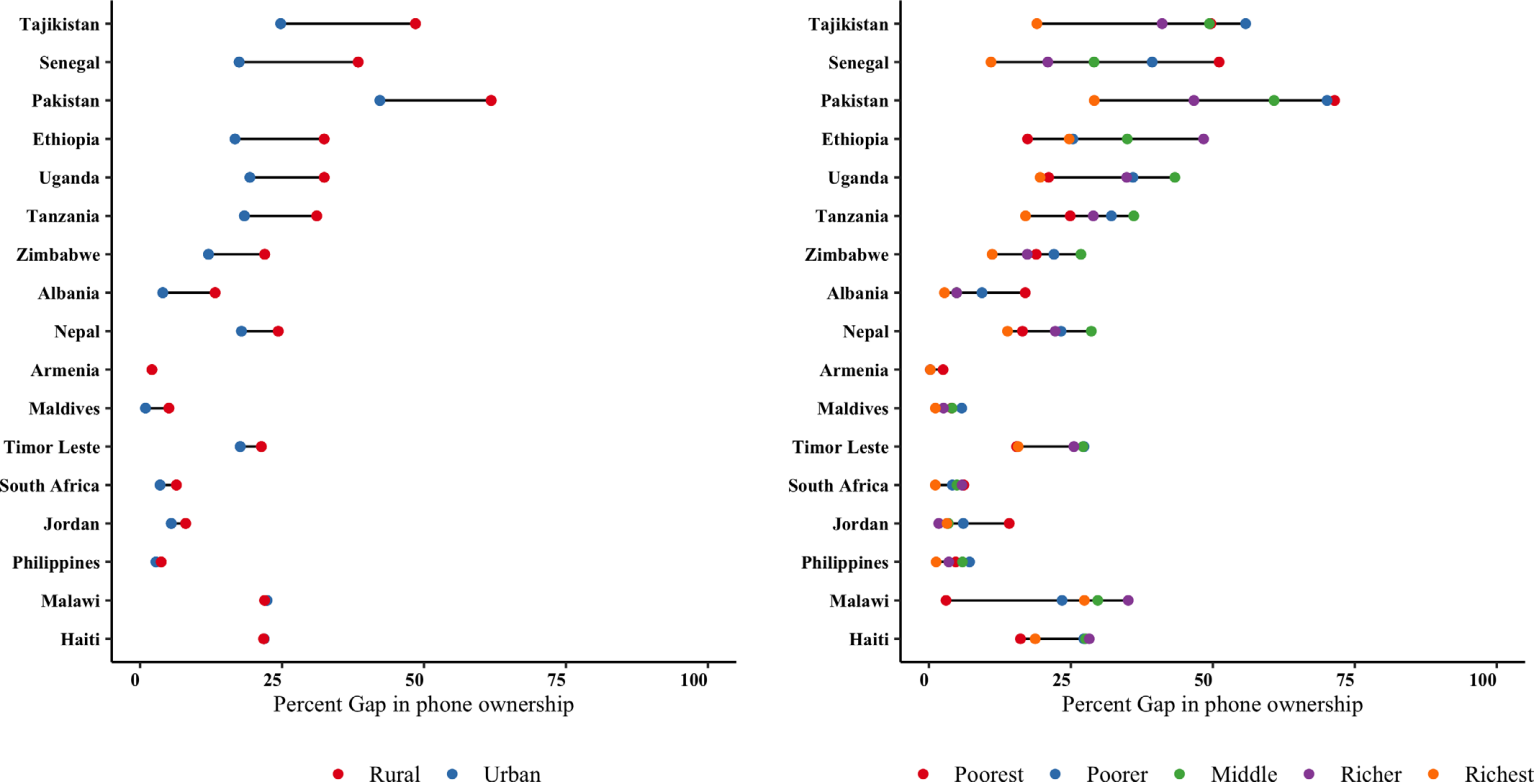
Equity dot plots of the gender gap in mobile phone ownership across 15 countries by residence and income. Country sequence is based on the magnitude of percentage difference between male and female mobile phone ownership by residence from greatest to smallest.

We also analysed the sociodemographic characteristics among men and women mobile phone owners (online supplementary table 1), the determinants of women’s phone ownership (online supplementary table 2), as well as the gender gap by residence and income strata by each country (figure 3). The odds of phone ownership was highest among women who are unmarried, older, wealthier, more educated, urban residents and with fewer children (after adjusting for wealth, age, education, parity, residence and martial status). In 10 out of the 15 countries, rural women bear the brunt of the gender gap in mobile phone ownership much more than urban women (figure 3). In many of these countries, men’s ownership is high regardless of residence, whereas women’s ownership varies substantially by residence. When looking at the gender gap in mobile phone ownership across income strata, it is substantially greater among poorer households than richer households in all but two countries (figure 3). The Maldives remained the most equitable with urban areas reporting a gender gap of 1% as compared with 2% in rural areas, while by income the richest have a 1% gender gap as compared with 4% in the poorest (3% divide).

## Women’s mobile phone ownership is associated with some but not all RMNCH improvements

**Figure 4:**
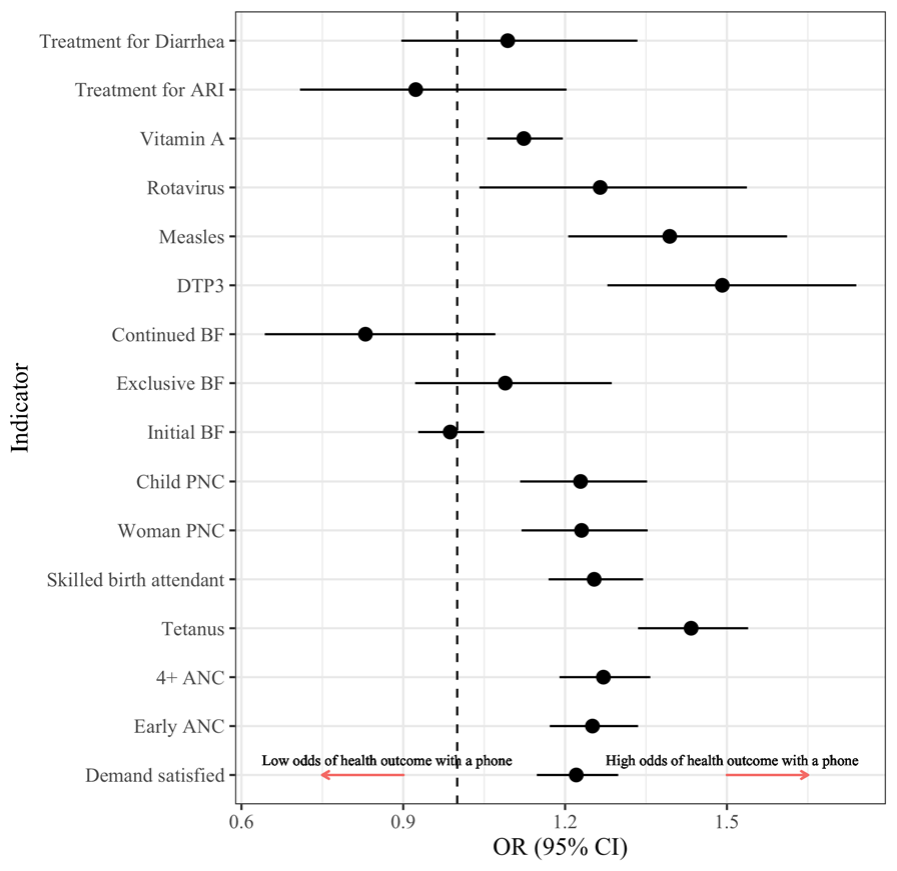
The odds* of adopting RMNCH care-seeking and practices among women who own a mobile phone. *ORs have been adjusted for sociodemographic characteristics presented in online supplementary table 1. ANC, antenatal care; ARI, acute respiratory infections; BF, breastfeeding; DTP-3, Diphtheria-tetanus-pertussis-3; PNC, postnatal care.

Figure 4 explores the association between women’s phone ownership and RMNCH health practices and care-seeking after adjusting for sociodemographic characteristics (indicators defined in online supplementary table 3; methods described in online supplementary file section 1; full results are presented in online supplementary tables 4–8). Women who owned a mobile phone had a higher odds of improved reproductive and maternal healthcare including demand satisfaction for family planning (22%), early antenatal care (ANC; 25%), 4+ ANC visits (27%), tetanus immunisation during pregnancy (43%), skilled attendance at birth (25%) and postnatal care for women (23%). Among child health services, phone ownership among women was similarly associated with higher odds of postnatal care for newborns (23%), vitamin A supplementation (12%), and immunisations including DTP3 (49%), measles (39%) and rotavirus (27%). Phone ownership among women was not significantly associated with breast feeding (initial, exclusive or continued) or care-seeking for acute respiratory infections or diarrhoea in children under 5 years of age.

**Figure 5:**
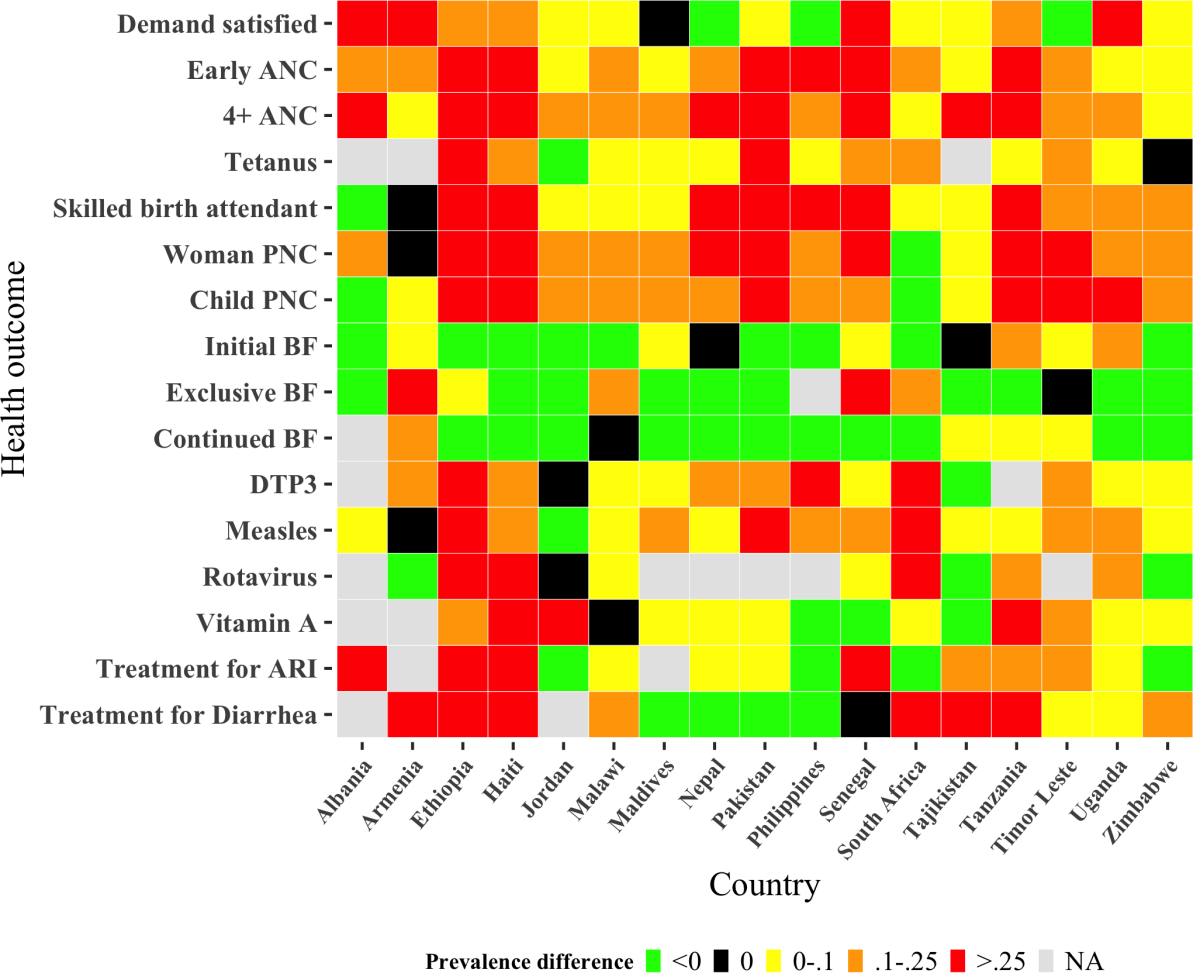
Estimates of the difference in RMNCH indicators between women with and without mobile phones by country. NA, not available.

In addition to overall estimates exploring the association between women’s mobile phone ownership and RMNCH care-seeking and practices, we used a heatmap to depict estimates of the relative differences in health indicators among women with and without a mobile phone by country (figure 5). Green-coloured cells denote lower adoption of a particular health practice by women who do own a mobile phone as compared with those who do not. By comparison, orange and red cells indicate higher adoption by women mobile phone owners as compared with women non-mobile phone owners. Black cells indicate no difference between women mobile phone owners and women non-phone owners for the specified indicator. Findings reinforce results above. In most of the 15 countries, care-seeking during pregnancy (early antenatal care, 4+ ANC), skilled birth attendance and postnatal care (maternal and child) was higher among women with a mobile phone versus those without. By comparison, the adoption of initial, exclusive and continued breast feeding was lower among women with a mobile phone as compared with those without a mobile phone in most countries.

**Figure 6:**
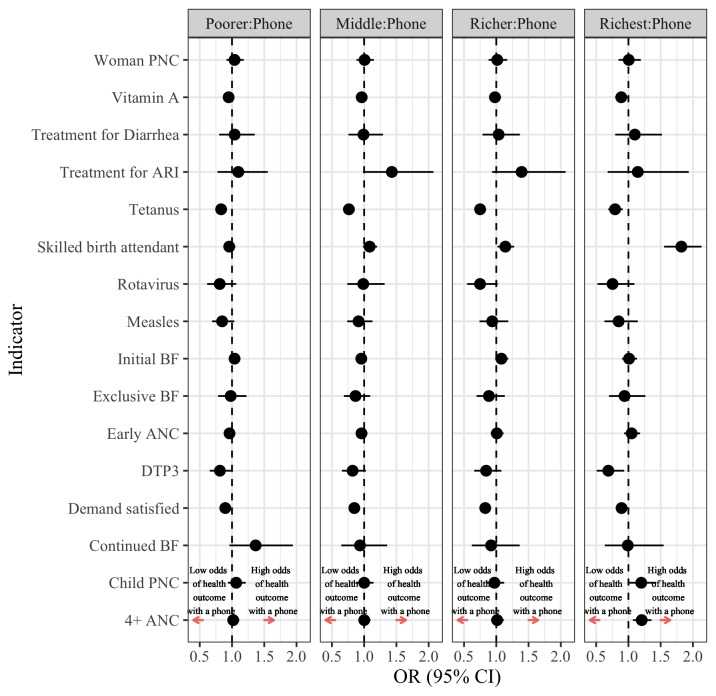
Linking women’s phone ownership with RMNCH care-seeking and practices across income strata.

## Among women phone owners, utilisation of RMNCH care-seeking and practices differ based on their income status

Figure 6 and online supplementary tables 4–8 consider the interaction of income with women’s phone ownership using the poorest as a reference case. Overall, a pro-poor bias was observed in demand satisfaction for family planning, tetanus immunisations during pregnancy, vitamin A supplementation and DTP3 immunisation. These findings are not surprising given that these programmes are often provided as part of campaigns or via community-based outreach, at little or no cost to users. In contrast, for utilisation of 4+ ANC, skilled attendance at birth and postnatal care for newborns, the opposite was observed as the richest women mobile phone owners have higher utilisation of services as compared with the poorest.

## Available data on the gendered nature of phone ownership is limited; a factor hampered by variations in the metrics used

Efforts to assess the relationship between mobile phones and RMNCH practices and care-seeking were hampered by limitations in available data. In the countries included, DHS contain indicators inquiring about available phones at a household level, women’s ownership of mobile phones, followed by a query on their use of phones for financial transactions. However, available indicators are limited and should be broadened to generate evidence on phone sharing, number of hours per day a phone is within reach, details on the characteristics of that phone including functionality, features, available credit, as well as phone use and the availability of mobile phone–based services which focus on different types of behaviour change. The latter is particularly important for facilitating understanding of differences between phone ownership and access to digital health services among phone owners. Beyond these indicators, efforts are needed to improve understanding of women’s digital literacy as a vital factor underpinning use.^21^ As mobile phones become more accessible and digital literacy increases, metrics for assessment will similarly need to evolve. In the interim, the analyses presented here represent a first step in understanding differentials in phone ownership and the effect women’s ownership may have on health outcomes.

## Conclusions

Despite increasing mobile phone ownership, inequalities persist by gender, geographical areas and sociodemographic characteristics. Study findings suggest that phone ownership among women may be associated with increased uptake of some RMNCH interventions, with a few notable exceptions. Women phone owners in the higher income strata were more likely to have 4+ ANC, skilled birth attendance and postnatal care for newborns (interventions that require significant healthcare seeking). In contrast, women mobile phone owners in the lowest income strata are less likely to have their demand for family planning satisfied, receive immunisations for tetanus and DTP3, and vitamin A supplementation (interventions that are proactively supplied by health services, often at the community level). Further research and improvements in digital health metrics and data collection is needed to improve understanding of and devise strategies for addressing gaps in women’s ownership and use of mobile phones so that it effectively leads to improved utilisation of different RMNCH interventions. The failure to do so could result in a worsening of health inequities among women and their families, as mobile phone penetration improves and health systems increasingly use mobile and other digital tools to improve health information and quality of care for those who can most easily access them versus those who need them the most.
