# Supplementary material for: Does women’s mobile phone ownership matter for health? Evidence from 15 countries
Source: BMJ Glob Health. 2020 May 17;5(5):e002524. doi: 10.1136/bmjgh-2020-002524 (PMC7245424; doi:10.1136/bmjgh-2020-002524)
Supplement: Supplementary data [file bmjgh-2020-002524supp001.pdf]

Supplementary File

Section 1. Summary of statistical methods undertaken

Data and sample

Data on phone ownership are presented for the 17 countries that have captured Demographic and Health Survey (DHS) data on and women's ownership of mobile phones and 15 countries that have captured data on men's ownership of mobile phones since 2015. Of the 17 countries which collected data on women's ownership, the Philippines and Tajikistan did not collect data on men's ownership and hence are excluded from gender gap analyses but retained in analyses exploring the association between women's phone ownership and RMNCH outcomes. The survey sample includes 242,619 women and 91,937 men from 204,214 households. Survey completion rates among eligible women ranged from 84% to 99%. The survey is designed as a two-stage sample design: In stage 1, the Primary Sampling Units (PSU) are villages in rural areas (selected with probability proportional to size); and Census Enumeration Blocks (CEB) in urban areas; in second stage, a random sample of households in each PSU or CEB is selected, respectively. For analysis, a cluster refers to either a PSU or a segment of a PSU selected at stage 1 of the survey. The data are hierarchical in nature with PSUs nested within districts, and districts are nested within states.

DHS Country	Survey Year(s)	Completion rate among eligible women
Albania	2017-2018	93%
Armenia	2015-2016	98%
Ethiopia	2016	95%
Haiti	2016-2017	99%
Jordan	2017-2018	99%
Malawi	2017	98%
Maldives	2016-2017	84%
Nepal	2016	98%
Pakistan	2017-2018	94%
Philippines	2017	98%
Senegal	2017	96%
South Africa	2016	86%
Tajikistan	2017	99%
Tanzania	2015-2016	97%
Timor Leste	2016	97%
Uganda	2016	97%
Zimbabwe	2015	96%

Analyses

Estimates are derived using the *survey* package version 3.34 in R and adjusted for survey design with appropriate weights provided as part of the DHS dataset using a robust variance estimator for the confidence intervals. The dot plots are based on the prevalence of the household mobile phone ownership and women's access to phone indicators, adjusted for the survey design with weights. The plots are constructed using the *ggplot2* package in R.

Multilevel logistic regression models were used the explore the association of RMNCH health outcomes with phone ownership. Models were adjusted for a range of socioeconomic and demographic characteristics, including age, educational attainment, household socio-economic status, residence, parity and marital status. The multilevel model is necessary since the data suggests considerable country level variance in the different health outcomes. The data have a hierarchical structure with women nested within clusters, which are in turn nested within and across countries. In the multilevel analysis, countries are the highest level (n=17), followed by clusters within countries and then individuals. The general form of the three level logistic regression model used may be expressed as

$$\log(P_{ijc}) = X_{ijc} * \beta + u_{jc} + v_c$$

where P_{ijs} is the probability of an outcome for an individual i, in the jth cluster in the cth country; X_{ijc} is the vector of covariates which may be defined at the individual, district or country level; β is the associated vector of regression parameter estimates; and the quantities, $u_{jc,}$ v_{kc} are the residuals at the cluster and country levels with normal distribution of mean zero and variances σ_u^2 , σ_v^2 respectively. The multilevel models were analyzed using the *lme4* package in R.

Section 2. Supplementary Tables and Figures.

Supplementary Table 1. Characteristics of men and women phone owners

	**Men Mobile Phone Ownership**				**Women Mobile Phone Ownership**			
	**Weighted Frequency**	**Percent**	**95% CI**		**Weighted Frequency**	**Percent**	**95% CI**	
**Wealth Index**								
Poorest	11,375	60%	59%	61%	22,714	44%	43%	45%
Poorer	12,972	70%	70%	71%	27,402	56%	56%	57%
Middle	13,708	75%	75%	76%	29,961	64%	63%	64%
Richer	14,079	81%	80%	82%	32,356	70%	70%	71%
Richest	16,956	90%	89%	90%	40,376	82%	81%	82%
**Age**								
15-19 years old	9,881	53%	52%	54%	19,737	45%	44%	45%
20-29 years old	21,818	82%	82%	83%	53,563	67%	66%	67%
30-39 years old	17,933	84%	83%	85%	44,707	68%	68%	69%
40-49 years old	19,458	76%	76%	77%	34,440	67%	67%	68%
**Education**								
No education	6,872	55%	54%	56%	14,133	34%	33%	35%
Primary	18,567	63%	63%	64%	34,689	48%	48%	49%
Secondary	32,352	84%	84%	84%	71,620	75%	75%	76%
Higher	11,299	97%	97%	98%	32,365	95%	95%	96%
**Parity**								
One child	NA	NA	NA	NA	24,240	70%	70%	71%
Two children	NA	NA	NA	NA	28,043	73%	73%	74%
>2 children	NA	NA	NA	NA	96,614	59%	58%	59%
**Residence**								
Urban	31,765	87%	87%	88%	77,328	79%	78%	79%
Rural	37,325	67%	67%	68%	75,481	52%	52%	53%
**Current Marital Status**								
Never in union	27,945	69%	68%	69%	48,891	62%	62%	63%
Married	39,865	81%	81%	82%	103,556	64%	63%	64%

Supplementary Table 2. Determinants of mobile phone ownership among women (n= 242,618)

Characteristics	Unadjusted Odds Ratio	p-value	Adjusted Odds Ratio	p-value
**Wealth Index**				
Poorest	1	--	1	--
Poorer	2.00 (1.93, 2.07)	<.001	1.79 (1.73, 1.85)	<.001
Middle	3.23 (3.11, 3.35)	<.001	2.57 (2.48, 2.67)	<.001
Richer	5.58 (5.37, 5.80)	<.001	3.81 (3.65, 3.97)	<.001
Richest	13.29 (12.71, 13.89)	<.001	7.21 (6.86, 7.59)	<.001
**Education**				
No education	1	--	1	--
Primary	1.69 (1.63, 1.74)	<.001	1.91 (1.84, 1.98)	<.001
Secondary	3.61 (3.49, 3.74)	<.001	4.15 (3.99, 4.31)	<.001
Higher	20.58 (19.34, 21.90)	<.001	14.16 (13.26, 15.12)	<.001
**Residence**				
Urban	1	--	1	--
Rural	0.28 (0.27, 0.30)	<.001	0.77 (0.74, 0.81)	<.001
**Parity**				
One child	1	--	1	--
Two children	1.00 (0.96, 1.04)	0.91	0.88 (0.85, 0.92)	<.001
>2 children	0.63 (0.61, 0.65)	<.001	0.75 (0.72, 0.78)	<.001
**Age**				
15-19 years old	1	--	1	--
20-29 years old	3.51 (3.41. 3.62)	<.001	3.75 (3.62, 3.89)	<.001
30-39 years old	3.47 (3.37, 3.59)	<.001	5.11 (4.92, 5.31)	<.001
40-49 years old	2.31 (2.24, 2.39)	<.001	3.96 (3.80, 4.12)	<.001
**Current Marital Status**				
Never in union	1	--	1	--
Married	1.41 (1.38, 1.45)	<.001	0.90 (0.87, 0.93)	<.001

Supplementary Table 3. RMNCH Health practice and care-seeking indicators examined in relation to mobile phone ownership

**Outcome**	**Description**
Demand satisfied by modern methods	Percentage of total demand for family planning that is satisfied by modern methods
4 +Antenatal care visits	Percentage of women with a birth in the last 5 years who had four or more antenatal care visits
Early Antenatal Care	Percentage of women with a birth in the last 5 years, who had a antenatal visit in the first 3 months of pregnancy.
Protected against neonatal tetanus	Percentage of women with a live birth in the 5 years preceding the survey whose most recent live birth was protected against neonatal tetanus:<br>• two tetanus toxoid injections during the pregnancy for her most recent live birth, or<br>• two or more injections (the last within 3 years of the most recent live birth), or<br>• three or more injections (the last within 5 years of the most recent live birth), or<br>• four or more injections (the last within 10 years of the most recent live birth), or<br>five or more injections at any time prior to the most recent live birth
Skilled birth attendance	Percentage of births in the 5 years preceding the survey that were assisted by a skilled provider.
Mother postnatal care	Percentage of women with a live birth in the 2 years preceding the survey who received a postnatal check during the first 2 days after giving birth.
Child postnatal care	Percentage of most recent live birth in the 2 years preceding the survey with a postnatal check for the newborn during the first 2 days after birth.

Initial Breastfeeding	Percentage of last-born children born in the past 2 years who started breastfeeding within one hour of birth.
Exclusive breastfeeding	Percent of children 0-5 months who were exclusively breastfed—not given anything else but breastmilk in the last 24 hours.
Continued breastfeeding at 1 year	Percentage of children currently breastfeeding, continuing breastfeeding at 1 year (12-15 months)
DTP3	Percentage of children age 12-23 months who received DTP3 at any time before the survey according to either vaccination card or mother's report.
Measles	Percentage of children age 12-23 months who received the first does of measles at any time before the survey according to either vaccination card or mother's report. *Notes: For Tajikistan and Armenia this is at 24-35 months
Rotavirus	Percentage of children age 12-23 months who received rotavirus vaccine at any time before the survey according to either vaccination card or mother's report.
Vitamin A Supplementation	Percentage of children age 6-59 months who received vitamin A supplements in the last six months.
ARI treatment	Percentage of children under age 5 with symptoms of ARI at any time in the 2 weeks preceding the survey for whom advice or treatment was sought.
Diarrheal care	Among children under age 5 who had diarrhea in the 2 weeks preceding the survey, percentage given ORS and zinc

Supplementary Table 4. Linking phone ownership, demand for modern contraception, and pregnancy care *

	Demand Satisfied				Early Antenatal Care visits				4+ Antenatal Care visits				Tetanus			
	OR	95% CI		P-value	OR	95%CI		P-value	OR	95%CI		P-value	OR	95%CI		P-value
**Mobile Phone Access**																
No mobile phone access	Reference				Reference				Reference				Reference			
Has Access	1.22	1.15	1.30	<.001	1.25	1.17	1.34	<.001	1.27	1.19	1.36	<.001	1.43	1.34	1.54	<.001
**Wealth**																
Poorest	Reference				Reference				Reference				Reference			
Poorer	1.25	1.18	1.33	<.001	1.21	1.13	1.28	<.001	1.23	1.16	1.30	<.001	1.20	1.12	1.28	<.001
Middle	1.41	1.32	1.51	<.001	1.32	1.23	1.41	<.001	1.42	1.33	1.52	<.001	1.31	1.21	1.41	<.001
Richer	1.46	1.36	1.57	<.001	1.44	1.33	1.55	<.001	1.59	1.48	1.71	<.001	1.39	1.27	1.52	<.001
Richest	1.48	1.34	1.63	<.001	1.81	1.63	2.01	<.001	1.92	1.73	2.12	<.001	1.57	1.38	1.78	<.001
**Wealth:Phone Interaction**																
Phone Access: Poorest	Reference				Reference				Reference				Reference			
Phone Access: Poorer	0.90	0.82	0.97	0.01	0.96	0.88	1.05	0.38	1.02	0.93	1.11	0.69	0.83	0.75	0.91	<.001
Phone Access: Middle	0.85	0.78	0.92	<.001	0.96	0.87	1.05	0.37	1.00	0.91	1.10	0.97	0.76	0.69	0.85	<.001
Phone Access: Richer	0.83	0.76	0.91	<.001	1.01	0.91	1.11	0.86	1.01	0.92	1.12	0.80	0.75	0.67	0.84	<.001
Phone Access: Richest	0.89	0.80	1.00	0.04	1.05	0.93	1.18	0.42	1.21	1.07	1.36	<.001	0.79	0.69	0.91	<.001
**Education**																
No education	Reference				Reference				Refernce				Reference			
Primary	1.17	1.12	1.22	<.001	1.38	1.31	1.45	<.001	1.44	1.37	1.50	<.001	1.48	1.40	1.56	<.001
Secondary	1.07	1.01	1.12	0.01	1.49	1.41	1.57	<.001	1.75	1.66	1.85	<.001	1.92	1.80	2.04	<.001
Higher	0.99	0.93	1.05	0.74	2.24	2.08	2.41	<.001	2.84	2.62	3.08	<.001	2.12	1.95	2.31	<.001
**Residence**																
Urban	Reference				Reference				Reference				Reference			

Rural	0.88	0.84	0.92	<.001	0.93	0.88	0.98	<.001	0.86	0.81	0.91	<.001	0.94	0.89	1.00	0.07
**Parity**																
One child	Reference				Reference				Reference				Reference			
Two Children	1.18	1.13	1.24	<.001	0.92	0.88	0.96	<.001	0.86	0.82	0.90	<.001	1.16	1.10	1.22	<.001
More than two children	1.15	1.11	1.21	<.001	0.73	0.70	0.77	<.001	0.72	0.69	0.76	<.001	1.28	1.21	1.34	<.001
**Age**																
15-19 years old	Reference				Reference				Reference				Reference			
20-29 years old	1.95	1.83	2.07	<.001	1.31	1.22	1.40	<.001	1.24	1.16	1.32	<.001	1.31	1.22	1.41	<.001
30-39 years old	2.34	2.20	2.50	<.001	1.43	1.33	1.55	<.001	1.39	1.29	1.50	<.001	1.26	1.16	1.37	<.001
40-49 years old	2.18	2.03	2.33	<.001	1.27	1.17	1.39	<.001	1.26	1.16	1.38	0.00	1.13	1.03	1.24	0.01
**Marital Status**																
Unmarried/not in union	Reference				Reference				Reference				Reference			
Married/In union	0.72	0.69	0.76	<.001	1.25	1.19	1.31	<.001	1.22	1.16	1.28	<.001	1.15	1.08	1.21	<.001

*Models are adjusted for survey round, phone access, wealth, education, residence, parity, age, and martial status

Supplementary Table 5. Linking phone ownership, skilled birth attendance, women and newborn postnatal care*

	Skilled Birth				Woman's Postnatal Care				Newborn Postnatal Care			
	OR	95%CI		P-value	OR	95%CI		P-value	OR	95%CI		P-value
**Mobile Phone Access**												
No mobile phone access	Reference				Reference				Reference			
Has Access	1.25	1.17	1.34	<.001	1.23	1.12	1.35	<.001	1.23	1.12	1.35	<.001
**Wealth**												
Poorest	Reference				Reference				Reference			
Poorer	1.41	1.33	1.50	<.001	1.25	1.15	1.35	<.001	1.24	1.14	1.34	<.001
Middle	1.75	1.64	1.87	<.001	1.44	1.31	1.58	<.001	1.47	1.34	1.61	<.001
Richer	2.17	2.00	2.36	<.001	1.67	1.50	1.86	<.001	1.77	1.59	1.97	<.001
Richest	2.92	2.56	3.34	<.001	2.19	1.89	2.54	<.001	1.90	1.64	2.21	<.001
**Wealth:Phone Interaction**												
Phone Access: Poorest	Reference				Reference				Reference			
Phone Access: Poorer	0.96	0.87	1.05	0.36	1.04	0.91	1.18	0.55	1.07	0.94	1.21	0.34
Phone Access: Middle	1.09	0.98	1.20	0.11	1.01	0.88	1.15	0.92	1.00	0.87	1.15	0.99
Phone Access: Richer	1.14	1.02	1.28	0.02	1.02	0.88	1.17	0.84	0.97	0.84	1.12	0.70
Phone Access: Richest	1.82	1.55	2.13	<.001	1.01	0.85	1.19	0.95	1.20	1.01	1.43	0.04
**Education**												
No education	Reference				Reference				Reference			
Primary	1.32	1.26	1.39	<.001	1.27	1.18	1.36	<.001	1.32	1.23	1.42	<.001
Secondary	2.08	1.96	2.21	<.001	1.74	1.61	1.89	<.001	1.80	1.66	1.95	<.001
Higher	4.98	4.41	5.62	<.001	2.60	2.32	2.91	<.001	2.30	2.06	2.58	<.001
**Residence**												
Urban	Reference				Reference				Reference			
Rural	0.42	0.39	0.46	<.001	0.76	0.71	0.82	<.001	0.72	0.67	0.78	<.001
**Parity**												
One child	Reference				Reference				Reference			

Two Children	0.59	0.55	0.63	<.001	0.81	0.76	0.86	<.001	0.81	0.76	0.87	<.001
More than two children	0.36	0.34	0.39	<.001	0.69	0.65	0.74	<.001	0.68	0.64	0.73	<.001
**Age**												
15-19 years old	Reference				Reference				Reference			
20-29 years old	1.08	1.00	1.18	0.06	1.18	1.08	1.28	<.001	1.12	1.03	1.22	0.01
30-39 years old	1.18	1.07	1.29	<.001	1.40	1.27	1.55	<.001	1.31	1.19	1.45	<.001
40-49 years old	1.09	0.99	1.21	0.09	1.38	1.21	1.57	<.001	1.30	1.14	1.49	<.001
**Marital Status**												
Unmarried/not in union	Reference				Reference				Reference			
Married/In union	1.10	1.04	1.17	<.001	1.04	0.96	1.12	0.31	1.07	0.99	1.16	0.08

*Models are adjusted for survey round, phone access, wealth, education, residence, parity, age, and martial status

Supplementary Table 6. Linking phone ownership and infant feeding practices*

	Initial Breastfeeding				Exclusive Breastfeeding				Continued Breastfeeding			
	OR	95%CI		P-value	OR	95%CI		P-value	OR	95%CI		P-value
**Mobile Phone Access**												
No mobile phone access	Reference				Reference				Reference			
Has Access	0.99	0.93	1.05	0.68	1.09	0.92	1.29	0.32	0.83	0.64	1.07	0.15
**Wealth**												
Poorest	Reference				Reference				Reference			
Poorer	0.97	0.92	1.03	0.33	1.01	0.87	1.16	0.94	0.80	0.63	1.03	0.08
Middle	1.04	0.98	1.11	0.22	1.03	0.87	1.20	0.76	0.91	0.69	1.21	0.51
Richer	0.94	0.87	1.01	0.10	0.85	0.71	1.01	0.07	0.82	0.60	1.12	0.22
Richest	0.97	0.87	1.08	0.58	0.84	0.65	1.08	0.17	0.66	0.45	0.98	0.04
**Wealth:Phone Interaction**												
Phone Access: Poorest					Reference				Reference			
Phone Access: Poorer	1.04	0.96	1.13	0.37	0.98	0.78	1.22	0.86	1.37	0.96	1.94	0.08
Phone Access: Middle	0.96	0.88	1.04	0.31	0.87	0.69	1.09	0.23	0.94	0.65	1.36	0.72
Phone Access: Richer	1.08	0.98	1.19	0.11	0.89	0.69	1.13	0.33	0.92	0.62	1.36	0.67
Phone Access: Richest	1.01	0.90	1.13	0.87	0.94	0.70	1.26	0.68	0.99	0.63	1.55	0.96
**Education**												
No education	Reference				Reference				Reference			
Primary	1.00	0.96	1.05	0.84	1.05	0.93	1.18	0.42	1.11	0.90	1.36	0.35
Secondary	1.03	0.97	1.09	0.30	0.98	0.85	1.12	0.75	0.99	0.79	1.25	0.96
Higher	0.96	0.89	1.03	0.23	0.90	0.75	1.08	0.24	0.82	0.62	1.07	0.15
**Residence**												
Urban	Reference				Reference				Reference			
Rural	0.93	0.88	0.98	0.01	0.97	0.87	1.08	0.57	1.17	1.00	1.37	0.05
**Parity**												
One child	Reference				Reference				Reference			

Two Children	1.15	1.10	1.20	<.001	0.94	0.84	1.06	0.33	1.09	0.92	1.30	0.32
More than two children	1.23	1.18	1.29	<.001	0.95	0.85	1.07	0.41	1.20	1.01	1.44	0.04
**Age**												
15-19 years old	Reference				Reference				Reference			
20-29 years old	1.00	0.94	1.07	0.94	1.17	1.02	1.34	0.03	1.15	0.89	1.48	0.29
30-39 years old	0.98	0.92	1.06	0.67	1.16	0.98	1.36	0.09	1.17	0.87	1.56	0.30
40-49 years old	0.97	0.89	1.06	0.50	1.32	1.04	1.68	0.02	1.05	0.72	1.54	0.80
**Marital Status**												
Unmarried/not in union	Reference				Reference				Reference			
Married/In union	1.02	0.97	1.07	0.35	1.02	0.88	1.19	0.75	1.31	1.05	1.64	0.02

*Models are adjusted for survey round, phone access, wealth, education, residence, parity, age, and martial status

Supplementary Table 7. Linking phone ownership and child health immunizations and vitamin A*

	DTP3				Measles				Rotavirus				Vitamin A			
	OR	95%CI		P-value	OR	95%CI		P-value	OR	95%CI		P-value	OR	95%CI		P-value
**Mobile Phone Access**																
No mobile phone access	Reference				Reference				Reference				Reference			
Has Access	1.49	1.28	1.74	<.001	1.39	1.21	1.61	<.001	1.27	1.04	1.54	0.02	1.12	1.06	1.20	<.001
**Wealth**																
Poorest	Reference				Reference				Reference				Reference			
Poorer	1.54	1.34	1.77	<.001	1.33	1.17	1.51	<.001	1.44	1.22	1.71	<.001	1.20	1.13	1.26	<.001
Middle	1.59	1.36	1.86	<.001	1.42	1.22	1.64	<.001	1.49	1.23	1.80	<.001	1.24	1.16	1.31	<.001
Richer	1.86	1.54	2.23	<.001	1.65	1.38	1.97	<.001	1.92	1.53	2.41	<.001	1.22	1.14	1.31	<.001
Richest	2.48	1.90	3.23	<.001	2.30	1.77	2.98	<.001	2.19	1.58	3.02	<.001	1.40	1.27	1.55	<.001
**Wealth:Phone Interaction**																
Phone Access: Poorest	Reference				Reference				Reference				Reference			
Phone Access: Poorer	0.81	0.65	1.01	0.06	0.85	0.69	1.04	0.10	0.81	0.61	1.07	0.13	0.95	0.87	1.03	0.20
Phone Access: Middle	0.82	0.66	1.03	0.08	0.91	0.74	1.13	0.40	0.99	0.74	1.32	0.92	0.96	0.88	1.05	0.38
Phone Access: Richer	0.84	0.66	1.08	0.17	0.94	0.74	1.19	0.59	0.75	0.55	1.02	0.07	0.98	0.89	1.07	0.65
Phone Access: Richest	0.69	0.51	0.93	0.02	0.85	0.63	1.15	0.28	0.75	0.52	1.09	0.14	0.89	0.79	1.00	0.04
**Education**																
No education	Reference				Reference				Reference				Reference			
Primary	1.75	1.55	1.96	<.001	1.61	1.44	1.79	<.001	1.63	1.41	1.89	<.001	1.24	1.19	1.30	<.001
Secondary	2.37	2.07	2.71	<.001	2.32	2.04	2.64	<.001	2.13	1.77	2.56	<.001	1.36	1.29	1.44	<.001
Higher	2.80	2.32	3.37	<.001	2.57	2.15	3.08	<.001	2.40	1.84	3.14	<.001	1.24	1.16	1.34	<.001
**Residence**																
Urban	Reference				Reference				Reference				Reference			
Rural	1.08	0.96	1.22	0.19	1.10	0.98	1.23	0.10	1.00	0.85	1.18	0.98	1.10	1.04	1.17	<.001
**Parity**																
One child	Reference				Reference				Reference				Reference			
Two Children	0.90	0.80	1.01	0.07	0.88	0.79	0.99	0.03	0.90	0.78	1.05	0.18	1.00	0.96	1.05	0.94
More than two children	0.79	0.70	0.89	<.001	0.72	0.64	0.81	<.001	0.84	0.72	0.98	0.02	0.96	0.92	1.01	0.11
**Age**																

15-19 years old	Reference				Reference				Reference				Reference			
20-29 years old	1.25	1.06	1.47	0.01	1.29	1.10	1.51	<.001	1.03	0.84	1.27	0.75	1.21	1.12	1.30	<.001
30-39 years old	1.55	1.30	1.87	<.001	1.51	1.26	1.80	<.001	1.24	0.98	1.56	0.07	1.39	1.29	1.51	<.001
40-49 years old	1.77	1.40	2.23	<.001	1.83	1.47	2.29	<.001	1.25	0.94	1.67	0.13	1.39	1.27	1.53	<.001
**Marital Status**																
Unmarried/not in union	Reference				Reference				Reference				Reference			
Married/In union	1.23	1.07	1.41	<.001	1.16	1.01	1.32	0.03	0.94	0.80	1.10	0.43	1.06	1.01	1.11	0.02

*Models are adjusted for survey round, phone access, wealth, education, residence, parity, age, and martial status

Supplementary Table 8. Linking phone ownership and careseeking for acute respiratory infections, and diarrhea treatment (ORS+Zinc)*

	ARI Careseeking				Diarrheal treatment			
	OR	95%CI		P-value	OR	95%CI		P-value
**Mobile Phone Access**								
No mobile phone access	Reference				Reference			
Has Access	0.92	0.71	1.20	0.55	1.09	0.90	1.33	0.38
**Wealth**								
Poorest	Reference				Reference			
Poorer	1.19	0.97	1.45	0.09	0.94	0.81	1.08	0.39
Middle	1.24	0.98	1.56	0.07	1.01	0.86	1.19	0.87
Richer	1.37	1.04	1.80	0.03	0.96	0.80	1.16	0.68
Richest	1.90	1.22	2.95	<.001	1.15	0.88	1.50	0.29
**Wealth:Phone Interaction**								
Phone Access: Poorest	Reference				Reference			
Phone Access: Poorer	1.10	0.78	1.55	0.60	1.04	0.80	1.35	0.77
Phone Access: Middle	1.43	0.98	2.07	0.06	0.99	0.76	1.29	0.94
Phone Access: Richer	1.39	0.93	2.07	0.10	1.04	0.79	1.37	0.80
Phone Access: Richest	1.14	0.68	1.93	0.61	1.10	0.79	1.52	0.57
**Education**								
No education	Reference				Reference			
Primary	1.19	1.00	1.42	0.05	1.12	0.99	1.27	0.07
Secondary	1.27	1.03	1.57	0.03	1.31	1.13	1.52	<.001
Higher	1.50	1.08	2.08	0.02	1.41	1.12	1.76	<.001
**Residence**								
Urban	Reference				Reference			
Rural	0.85	0.71	1.01	0.06	1.00	0.88	1.14	0.99
**Parity**								
One child	Reference				Reference			
Two Children	0.98	0.80	1.20	0.85	0.97	0.85	1.10	0.59
More than two children	0.81	0.67	1.00	0.05	0.88	0.78	1.00	0.06
**Age**								
15-19 years old	Reference				Reference			
20-29 years old	1.14	0.84	1.55	0.39	1.26	1.06	1.50	0.01
30-39 years old	1.31	0.94	1.82	0.11	1.34	1.10	1.64	<.001
40-49 years old	1.48	1.01	2.17	0.05	1.35	1.04	1.74	0.02
**Marital Status**								
Unmarried/not in union	Reference				Reference			
Married/In union	0.97	0.78	1.22	0.80	1.10	0.96	1.26	0.16

*Models are adjusted for survey round, phone access, wealth, education, residence, parity, age, and martial status
